# Maca extracts regulate glucose and lipid metabolism in insulin‐resistant HepG2 cells via the PI3K/AKT signalling pathway

**DOI:** 10.1002/fsn3.2246

**Published:** 2021-03-29

**Authors:** Aimin Li, Jia Liu, Fangli Ding, Xiaolei Wu, Cong Pan, Qing Wang, Ming Gao, Shenglin Duan, Xiaofeng Han, Kai Xia, Shiwei Liu, Yimin Wu, Zhiqiao Zhou, Xi Zhang, Xiao‐Dong Gao

**Affiliations:** ^1^ Key Laboratory of Carbohydrate Chemistry and Biotechnology, Ministry of Education, School of Biotechnology Jiangnan University Wuxi China; ^2^ New Era Health Industry (Group) CO., Ltd. Beijing China; ^3^ Beijing Key laboratory of the Innovative Development of Functional Staple and the Nutritional Intervention for Chronic Disease Beijing China; ^4^ China National Research Institute of Food and Fermentation Industries Beijing China; ^5^ Shimadzu(China) Co. Ltd. Beijing China

**Keywords:** *AKT*, glucose and lipid metabolism, Maca ethanol extracts, *PI3K*

## Abstract

This work focused on the separation of the active ingredients of maca (*Lepidium meyenii* Walpers) and evaluated the antioxidative capability of these components with effects on improving glucose and lipid metabolism in insulin‐resistant HepG2 cells. DPPH free radical scavenging and reducing power assays were used to evaluate the antioxidant activity of maca extracts. An insulin‐resistant HepG2 cell model induced by glucose, fructose, oleic acid, and palmitic acid was adopted to investigate the effects of maca extracts on regulating glucose and lipid metabolism in this study. LC‐MS/MS was then used for determination of the maca n‐butanol (NBT) subfraction. The results showed that maca ethanol extract and subfractions of this extract exhibited certain antioxidant capacity. Furthermore, the NBT subfraction reversed the disorders in glucose and lipid metabolism in insulin‐resistant HepG2 cells and significantly increased the mRNA expression of phosphoinositide 3‐kinases (*PI3K*) and *AKT* in insulin‐resistant HepG2 cells in a dose‐dependent manner. In addition, the LC‐MS/MS results showed that the NBT subfraction contained many active ingredients. Overall, this study suggests that the NBT subfraction of the ethanol extract rich in glucosinolates modulates insulin resistance via *PI3K*/*AKT* activation in insulin‐resistant HepG2 cells and might exert potentially beneficial effects in improving or treating glucose and lipid metabolic disorders.

## Graphical abstract

This work focused on the separation of Maca (*Lepidium meyenii* Walp.) active ingredients, and evaluated the antioxidative capability of these components with the effects on improving glucose and lipid metabolism in insulin resistant HepG2 cells.

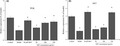

## 1 INTRODUCTION

Diabetes mellitus refers to a group of chronic metabolic diseases characterized by high‐blood sugar and has become a worldwide epidemic. According to the latest report published by the International Diabetes Federation in 2019, 463 million adults (aged 20–79 years) are currently living with diabetes mellitus worldwide, and it has been estimated that 578.4 million people will be suffering from diabetes mellitus and complications in 2030 (International Diabetes Federation, 2019). Type 2 diabetes mellitus, which is the most common, accounting for approximately 90% of cases, and harmful to human health (Shi & Hu, 2014), is characterized by insulin resistance (Castillo et al., 2012) and might cause many severe chronic complications, such as cardiovascular disease, neuropathy, nephropathy, and retinopathy (Schram et al., 2014). Therefore, effective prevention and treatment is critical for type 2 diabetes. At present, the clinical drugs for type 2 diabetes treatment mainly include metformin, alpha glucosidase inhibitors, sulfonylureas, thiazolidinediones, and meglitinides, and all of these drugs have a number of side effects. Therefore, the identification of a safer and more effective method for alleviating type 2 diabetes is necessary. Type 2 diabetes is closely related to lifestyle, and dietary intervention is considered an effective alternative method for alleviating or treating diabetes. The development of functional foods that can alleviate insulin resistance is of great importance in clinical settings and would have fewer side effects than drugs. In recent years, natural products have been widely investigated, and the active ingredients in some edible plants have been proven to enhance the metabolism of glucose and lipids through free radical scavenging and inflammation improvements (Eid et al., 2017; Shen et al., 2019).

*Lepidium meyenii* Walpers, which is known as maca, is widely cultivated in the central highlands of the Peruvian Andes and China and was approved as a novel food by the Chinese government in 2011 (Hao et al., 2014). Maca has attracted widespread attention due to its function in sexual improvement, fertility enhancement, antioxidant activity, antifatigue activity, and memory impairment (Choi et al., 2012; Uchiyama et al., 2014), which are closely related to the natural active ingredients contained in maca. Maca is not only rich in polysaccharides, tannins, fat oils, branched amino acids, and other components but also contains some secondary metabolites, such as glucosinolates, macaamide, macacene, glucosinolates, flavonoids, terpenes, and sterols. Some studies have found that feeding a certain amount of maca to diabetic rats could decrease the levels of lipids and glucose in the blood (Vecera et al., 2007). The report showed the protective effects of maca against type 2 diabetes, but the specific mechanism through which maca regulates insulin resistance is not very clear. Therefore, exploring the mechanism through which maca alleviates insulin resistance is of great importance. The phosphoinositide 3‐kinase (*PI3K*)/*AKT* and *AMP*‐activated protein kinase (*AMPK*) signalling pathways play critical roles in regulating glucose metabolism in insulin‐resistant cells (Hardie, 2004). *PI3K*/*AKT* is a major downstream molecular pathway of insulin (Hardie, 2004). The activation of AMPK switches off anabolic pathways that consume adenosine triphosphate (ATP), such as glycogen, and switches on catabolic pathways that generate ATP, such as glycolysis (Steinberg & Kemp, 2009).

In the present study, we investigated the effects of different subfraction from maca extracts in regulating glucose metabolism in insulin‐resistant HepG2 cells and explored the active ingredients that might play a key role in regulating insulin resistance. The key genes and signal transduction pathways involved in improving glucose metabolism were also analyzed in this study. We obtained the n‐butanol subfraction of maca ethanol extract and provided the first clarification of its effects on regulating glucose and lipid metabolism in insulin‐resistant HepG2 cells. More importantly, this work can be helpful for further understanding the underlying mechanism through which maca regulates glucose and lipid metabolism.

## 2 MATERIALS AND METHODS

### 2.1 Chemicals and drugs

Black maca was purchased from a farm in Yunnan Province of New Era Health Industry (Group) Co., Ltd. Phosphate‐buffered saline (PBS), Hank's balanced salt solution (HBSS), bovine serum, Dulbecco's modified Eagle's medium (DMEM), and glucose‐free DMEM were purchased from Gibco Life Technologies; oleic acid (OA), palmitic acid (PA), 1,1‐diphenyl‐2‐picrylhydrazyl (DPPH, 90%), dimethyl sulfoxide (DMSO), 3‐[4,5‐dimethylthiazol‐2‐yl]‐2,5‐diphenylterazolium bromide (MTT), oil red O staining agent, trypsin, rutin standard, and macaamide standard were purchased from Sigma; fatty acid‐free bovine serum albumin (BSA) was provided by WAKO; RIPA lysate and TG, glucose assay and glycogen assay kits were procured from Nanjing Jiancheng Bioengineering Institute; and ethanol, petroleum ether, ethyl acetate, and *n*‐butanol were purchased from Sinopharm Chemical Reagent Co., Ltd. All other chemicals and solvents, all of which were of analytical grade, were obtained from China.

### 2.2 Fractional extraction of maca

Dried black maca was powdered using a Philips Juicer (Zhuhai Special Economic Zone Philips Home Appliances Co., Ltd.) and sifted through a 100‐mesh sieve. Maca powder was extracted twice with 75% (*v/v*) ethanol at a solid–liquid ratio of 1:15 (*w/v*) under sonication for 30 min at 50°C. The mixture was then centrifuged at 400 *g*, and the supernatant was collected. Ethanol was removed by decompression distillation at 40°C using an IKA^®^ RV 10 instrument (Zhengzhou Changchengke Industry and Trade Co., Ltd), and maca ethanol extract (MEE) was collected. MEE was further extracted successively with petroleum ether, ethyl acetate, and water‐saturated *n*‐butanol, and the extracts remaining in the aqueous phase were dried using an LGJ‐18 Vacuum freeze dryer (Beijing SongyuanHuaxing Technology Development Co., Ltd.). Finally, maca subfractions of the petroleum ether phase (PE), maca ethyl acetate phase (EA), maca n‐butanol phase (NBT), and maca aqueous phase (WAT) were obtained.

### 2.3 Antioxidant capacity assay

The scavenging capacity of free radicals was evaluated using the DPPH test as described previously for maca extracts (Zevallos‐Concha et al., 2016). The ability of maca extracts to scavenge DPPH radicals was measured by the discoloration of the solution (Mensor et al., 2001). Briefly, 1.0 ml of the extracts at concentrations of 0–5.0 mg/ml were mixed with 1.0 ml of DPPH methanol solution. The mixtures were then incubated for 30 min in the dark at room temperature, and the absorbance was measured with a spectrophotometer at 517 nm. The reading was performed against a blank without maca extract. The data are expressed as percentages of the scavenging capacity of free radicals determined using the DPPH test.

The ferric reducing antioxidant power (FRAP) assay was used to measure the total antioxidant power (Benzie & Szeto, 1999). In brief, 1.0 ml of 0.2 M PBS at pH 6.6, 1.0 ml of 1% potassium ferricyanide solution and 1.0 ml of extract solution were added to 5.0‐ml test tubes, and the mixture was shaken and immediately heated in a water bath at 50°C for 20 min. Subsequently, 1.0 ml of 10% trichloroacetic acid was added, and the test tube was cooled to room temperature and then shaken. The mixed solution was centrifuged for 10 min at 200 *g*, and 2.0 ml of the supernatant was then removed and mixed with 3.0 ml of distilled water and 0.2 ml of 0.1% ferric chloride. The mixture was allowed to stand for 10 min, and the absorbance was measured at 725 nm using distilled water as the control treatment.

### 2.4 Cell culture and sample preparation

HepG2 cells were supplied by Peking Union Medical College Hospital, Chinese Academy of Medical Sciences. The culture was maintained in 10% FBS in DMEM containing 1% antibiotic antimycotic solution (100×) at 37°C in an incubator with a humidified atmosphere and 5% CO_2_. The extracts were dissolved in DMSO, diluted with DMEM, and then filtered through a 0.22‐μm Millipore membrane prior to their use for the treatment of HepG2 cells. The final concentration of DMSO did not exceed 0.1% of the culture media in all the experiments (Liu et al., 2014).

### 2.5 Cell viability assay

HepG2 cells (1 × 10^5^ cells/well) were grown in 96‐well plates with different concentrations of the maca extracts for 24 hr. After 24 hr, the medium was replaced with 20 μl of MTT solution (5 mg/ml), and the cells were incubated for another 4 hr at 37°C. The medium containing MTT was then carefully removed to avoid disturbing the formation of mazan crystals. DMSO was added, and the absorbance at 490 nm was recorded using a Spectro Max i3 microplate reader (MD Electronics). The cell viability was expressed as a percentage relative to that of the control group without maca extracts.

### 2.6 Establishment of insulin‐resistant HepG2 cell line model

Previous studies have shown that free fatty acids, such as oleic acid and palmitic acid, can induce fat accumulation in HepG2 cells (Chen & Wu, 2005; Marinković et al., 2019), and the combination of glucose, fructose, and free fatty acids (FFAs) could successfully induce metabolic disorders in HepG2 cells, including dyslipidemia, insulin resistance, hyperuricemia, and oxidative stress (Zhao et al., 2016). Therefore, a cell model that featured insulin resistance was established by treatment with glucose (G) and fructose (F) plus FFA in this study, and the best combination was selected based on the analysis of several different treatment groups. First, the cells (3.5 × 10^6^) were grown in a 96‐well plate in high‐glucose DMEM with 10% FBS. After 24 hr, the medium was replaced with DMEM containing glucose, fructose and FFAs at different concentrations (10G + 15F + 1FFA, 10G + 15F + 2FFA, 10G + 25F + 1FFA, 10G + 25F + 2FFA, 20G + 15F + 1FFA, 20G + 15F + 2FFA, 20G + 25F + 1FFA, 20G + 25F + 2FFA), and cells grown in serum‐free DMEM containing 1% BSA served as the control group (Li et al., 2018). The group name 20G + 15 F + 1FFA indicates treatment with 20 mM glucose, 15 mM fructose and 1 mM FFA, which is a mixture of oleic acid, palmitic acid and BSA at a molar ratio of 4.4:2.2:1.

### 2.7 Glucose uptake assay

After 24 hr of treatment with the maca extracts, HepG2 cells were washed and cultured in DMEM containing 12.5 mM glucose. The glucose concentration in the medium was determined using a glucose assay kit (Nanjing Jiancheng Bioengineering Institute). The amount of glucose consumption was calculated using the initial glucose level and the glucose level remaining in the medium (So et al., 2016; Zhang et al., 2017). In addition, a standard antidiabetic drug, metformin (30 μM), was used as a positive control.

### 2.8 Intracellular glycogen assay

The culture medium was removed after 24 hr of treatment with the maca extracts, and the HepG2 cells were then washed with PBS and dissolved in RIPA lysis buffer. The glycogen concentration in the cell was determined using a glycogen assay kit.

### 2.9 Intracellular and supernatant TG assay

The culture medium was removed after 24 hr of treatment with the maca extracts, and the intracellular and supernatant TG levels were detected using the TG kit according to the instructions (Zhang et al., 2013).

### 2.10 Oil Red O staining

The cells were washed with PBS and fixed with 75% freshly prepared ethanol for 10 min. The fixed cells were washed three times with water and then stained with Oil Red O solution for 10 min. The excess stain was removed, and the stained cells were dried completely, observed under a light microscope and photographed (Yoon et al., 2013).

### 2.11 LC‐MS/MS analysis

The NBT fraction was analyzed using an LCMS‐9030 (Shimadzu) equipped with a Shim‐pack GIST‐C18 column (2 μm, 2.1 mm × 100 mm). Water containing 0.1% formic acid (A) and acetonitrile (B) were used as the elution solvents. The elution program was the following: 0–2 min, 5% B; and 2–10 min, 5%–25% B. The flow rate was 0.2 μl/min, and the sample loading was 2 μl. A full MS scan was used with *m*/*z* 50–1000. The MS parameters of MS were set as follows: spray voltage, 3.3 kV; capillary temperature, 320°C; heater temperature, 350°C; auxiliary gas flow, 8.0 L/min; sheath gas flow rate, 32.0 L/min; sweep gas, 4.0 L/min; and S‐lens RF level, 50%.

### 2.12 Quantitative real‐time PCR (qRT‐PCR)

Total RNA from HepG2 cells was prepared with TRIzol (Invitrogen) and an RNeasy kit following the manufacturer's instructions. cDNA was synthesized using a QuantiTect Reverse Transcription kit (Qiagen) with 2 μg of total RNA and amplified using SYBR Premix Ex Taq and gene‐specific primers with a Takara Thermal Cycler Dice Real‐Time system (40 cycles of 95°C for 5 s, 58°C for 10 s, and 72°C for 20 s). The primers included *PI3K* (forward 5′‐*CTATCCAGACCAGTACGTTCG*‐3′, and reverse 5′‐*GAGGGCACAATCAAGAAAAGG*‐3′), *AKT* (forward 5′‐*TCTATGGCGCTGAGATTGTG*‐3′, and reverse 5′‐*TCTTAATGTGCCCGTCCTTG*‐3′), and GAPDH (forward 5′‐*TTCTGGGATACACGGAGCAC*‐3′, and reverse 5′‐*TACCAGCACCAGCGTCAAAG*‐3′). Once the reactions were completed, a melting curve was constructed to confirm that the amplification was successful. The data were analyzed using Opticon Monitor software (ver. 3.1; Bio‐Rad Laboratories).

### 2.13 Statistical analysis

The data are presented as the means ± standard deviations (SDs) from at least three replicates of each sample. The statistical analyses were performed using SPSS 20.0 software (SPSS Inc.). The data were analyzed by one‐way ANOVA, and *p* <.05 indicated significant differences.

## 3 RESULTS

### 3.1 Results from the screening for antioxidant capacity in vitro

**FIGURE 1:**
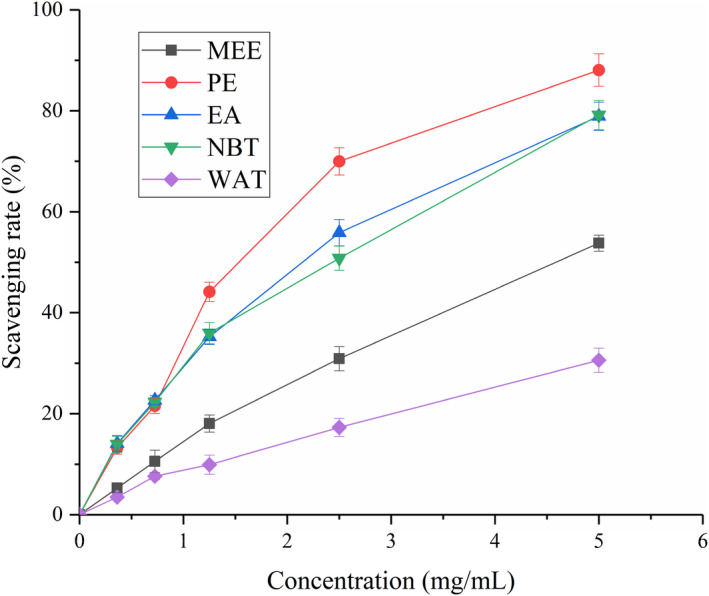
DPPH inhibition rates obtained with different concentrations of the samples. Subfractions: MEE, maca ethanol extract; PE, petroleum ether; EA, ethyl acetate; NBT, n‐butanol; and WAT, water

As depicted in Figure 1, increases in the concentration of maca extracts in the range of 0.2–5 mg/ml gradually increased the inhibition rate of DPPH. In addition, PE exhibited the most significant DPPH inhibition ability (IC 50 = 1.68 mg/ml). The radical scavenging activity of PE might be due to the presence of macaamide. Table 1 shows the total antioxidant ability of maca extracts compared with that of the standard rutin. Different maca extracts showed different antioxidant activities, ranging from 0.77 ± 0.07 for WAT to 3.21 ± 0.04 mg rutin/g for the NBT fraction. These results indicated that maca alcohol extract had potential antioxidant capacity, which was consistent with previous research (Feng et al., 2010).

**TABLE 1:** Antioxidant capacity of different Maca extracts in vitro

Maca extracts	DPPH scavenging^a^ IC_50_ (mg/ml)	mg rutin/g maca extract^b^
MEE	4.53	1.91 ± 0.05
PE subfraction	1.68	0.96 ± 0.22
EA subfraction	2.14	0.41 ± 0.08
NBT subfraction	2.67	3.21 ± 0.04
WAT subfraction	4.84	0.77 ± 0.07

Abbreviations: EA, ethyl acetate; MEE, maca ethanol extract; NBT, *n*‐butanol; PE, petroleum ether; WAT, water.

^a^ DPPH radical scavenging ability of different maca extracts.

^b^ Total antioxidant power of different maca extracts measured using the FRAP assay.

### 3.2 Effect of MEE and subfractions of MEE on cell proliferation

**FIGURE 2:**
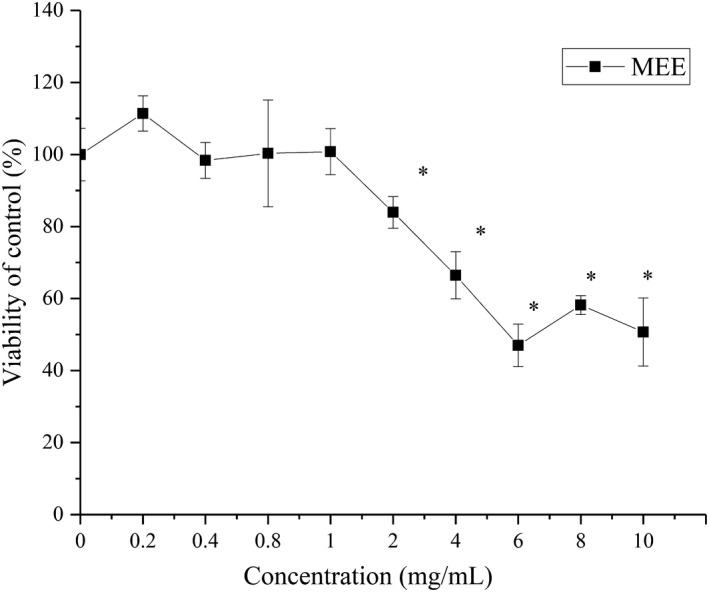
Effects of maca ethanol extract on cell viability. MEE: maca ethanol extract, *p < .05 compared with the control group

The appropriate concentrations of subfractions of maca ethanol extract for cellular treatment were determined using the MTT assay. As shown in Figure 2, maca ethanol extract did not influence the viability of HepG2 cells at concentrations of 0–1 mg/ml. However, an MEE concentration higher than 1 mg/ml significantly decreased the cell viability rate.

**FIGURE 3:**
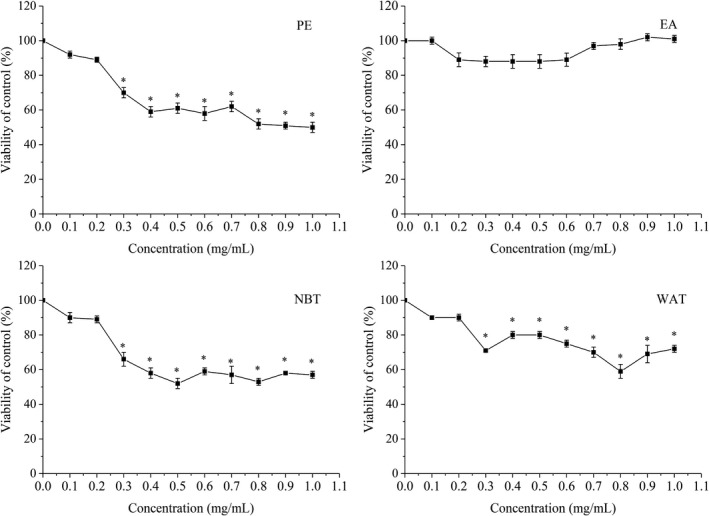
Effects of maca ethanol extract subfractions on cell viability. Subfractions: PE, petroleum ether; EA, ethyl acetate; NBT, n‐butanol; and WAT, water. *p < .05 compared with the control group

The PE, EA, NBT, and WAT fractions (0.1–0.2 mg/ml) did not influence the viability of HepG2 cells (Figure 3). At a concentration of at least 0.3 mg/ml, the PE, NBT, and WAT fractions significantly inhibited cell growth (*p* < .05). Thus, 0.2 mg/ml was selected as the highest concentration used for further experiments.

### 3.3 Establishment of a metabolic disorder model of HepG2 cells

**FIGURE 4:**
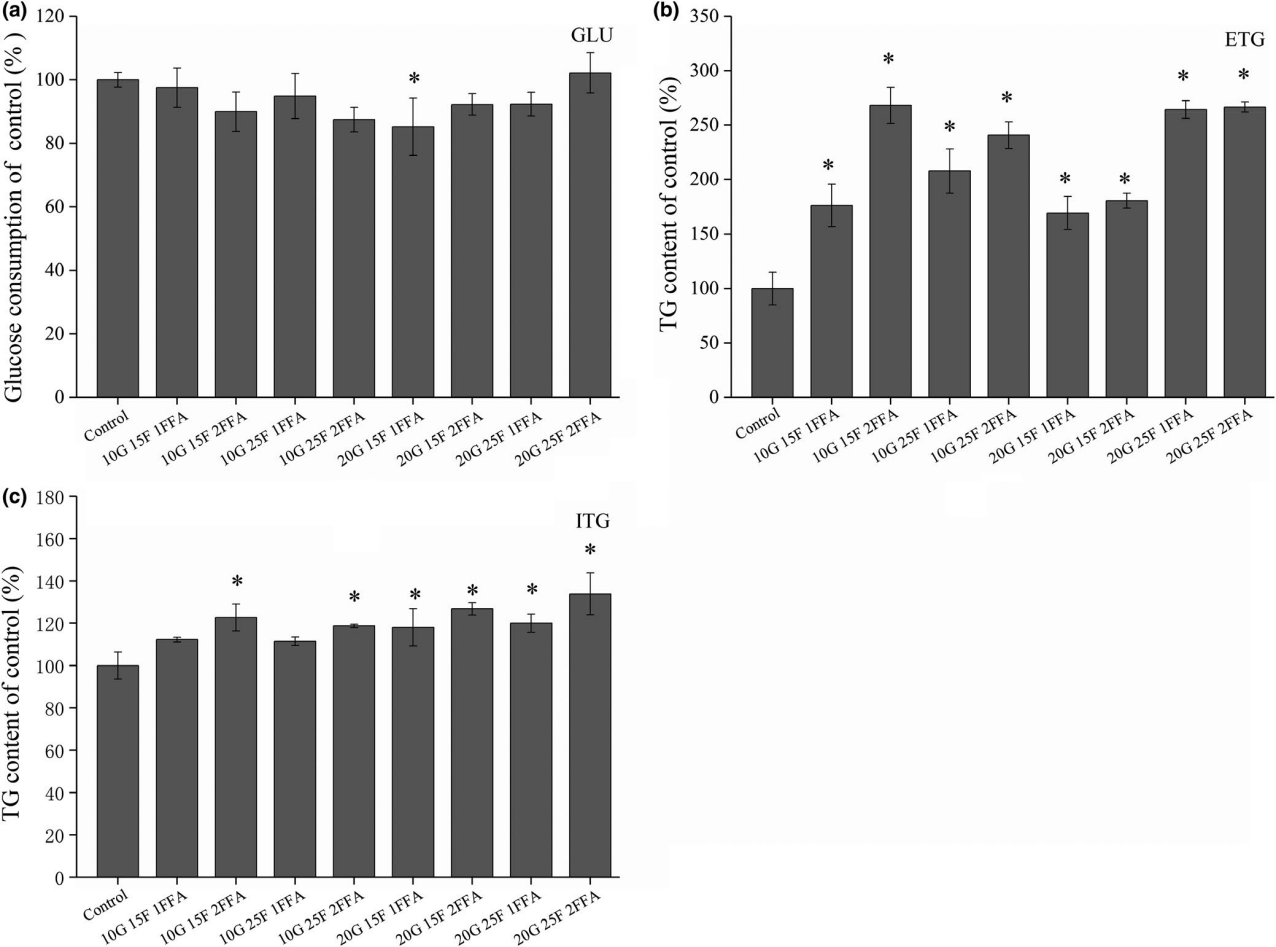
Establishment of the insulin resistance cell model. (a), Effect of supplementation with glucose, fructose and free fatty acids on glucose consumption in HepG2 cells. (b), Effect of supplementation with glucose, fructose and free fatty acids on the contents of extracellular triglycerides. (c) Effect of supplementation with glucose, fructose and free fatty acids on the contents of intracellular triglycerides. GLU, glucose consumption; ITG, intracellular triglyceride; ETG, extracellular triglyceride. *p < .05 compared with the control group

As presented in Figure 4, the glucose consumption of extracellular glucose was significantly lower in the 20G + 15F + 1FFA‐treated group than in the control group (*p* < .05). In addition, the extracellular triglyceride (ETG) and intracellular triglyceride (ITG) levels were significantly increased in almost all the treatment groups (*p* < .05). In particular, the ETG and ITG contents of the 20G + 15F + 1FFA‐treated group were approximately 2.6‐ and 1.2‐fold higher than those in the control group, respectively. Considering the three above‐mentioned indicators, the 20G + 15F + 1FFA group was selected to establish the model of glucose and fructose metabolism disorder.

**FIGURE 5:**
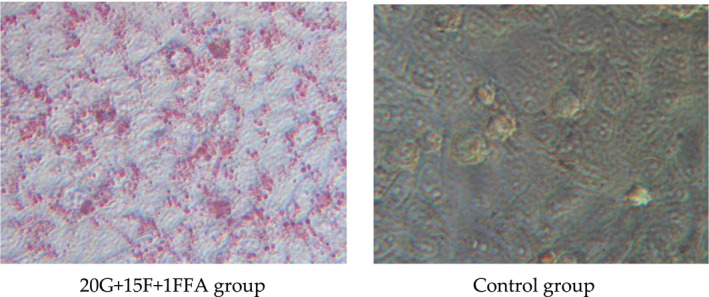
Images of Oil red O‐stained cells. 20G + 15 F + 1FFA: cells treated with 20 mM glucose, 15 mM fructose and 1 mM FFA; Control group: untreated cells

Furthermore, the cell model was verified by staining, and images of Oil red O‐stained cells treated with 20G + 15F + 1FFA are shown in Figure 5. Oleic acid clearly induced an increase in the cellular lipid content, as determined by a higher number of red lipid globules inside the treated cells than in the control cells. All of the above results indicated that the cell model of glucose and lipid metabolic disorder was successfully established.

### 3.4 Effect of maca ethanol extract on glucose and lipid homeostasis

**FIGURE 6:**
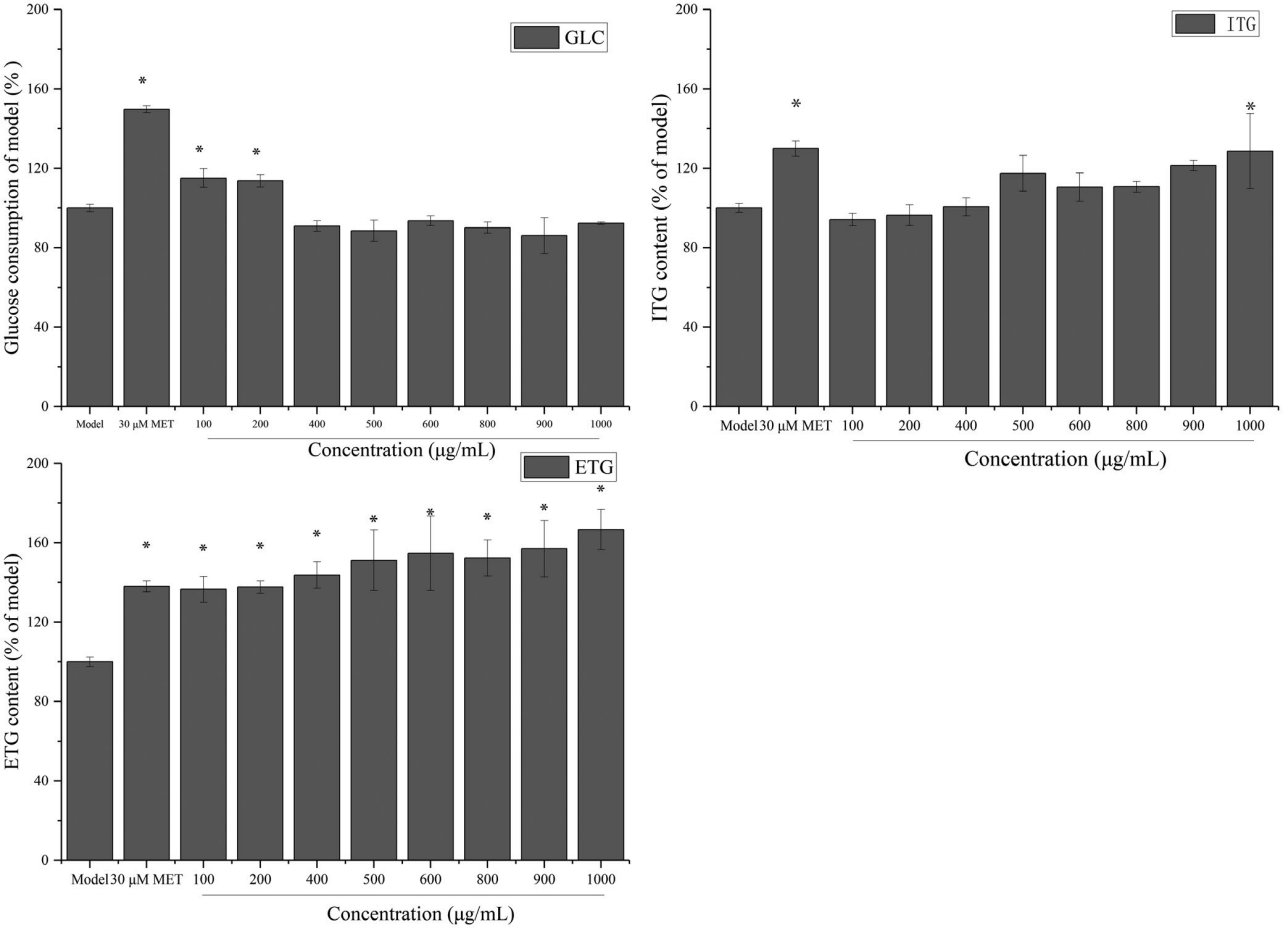
Maca ethanol extract regulates glucose consumption in HepG2 cells. GLU, glucose consumption; ITG, intracellular triglyceride; ETG, extracellular triglyceride. *p < .05 compared with the model group

The effects of maca ethanol extract on regulating glucose and lipid metabolism are shown in Figure 6. The level of glucose consumption from the cell culture medium first increased and then decreased, and significant differences (*p* < .05) were found at concentrations of 100–200 μg/ml, which indicated that maca ethanol extract at concentrations of 100–200 μg/ml could promote glucose consumption. The intracellular TG level was significantly increased with the high concentration of maca ethanol extract, and the extracellular TG content in the group treated with maca ethanol extract at a concentration of 1,000 μg/ml was 65% higher than that in the model group. The results suggested that maca ethanol extract might improve disorders in glucose and lipid metabolism.

### 3.5 Effect of subfractions of maca ethanol extract on glucose and lipid homeostasis

**FIGURE 7:**
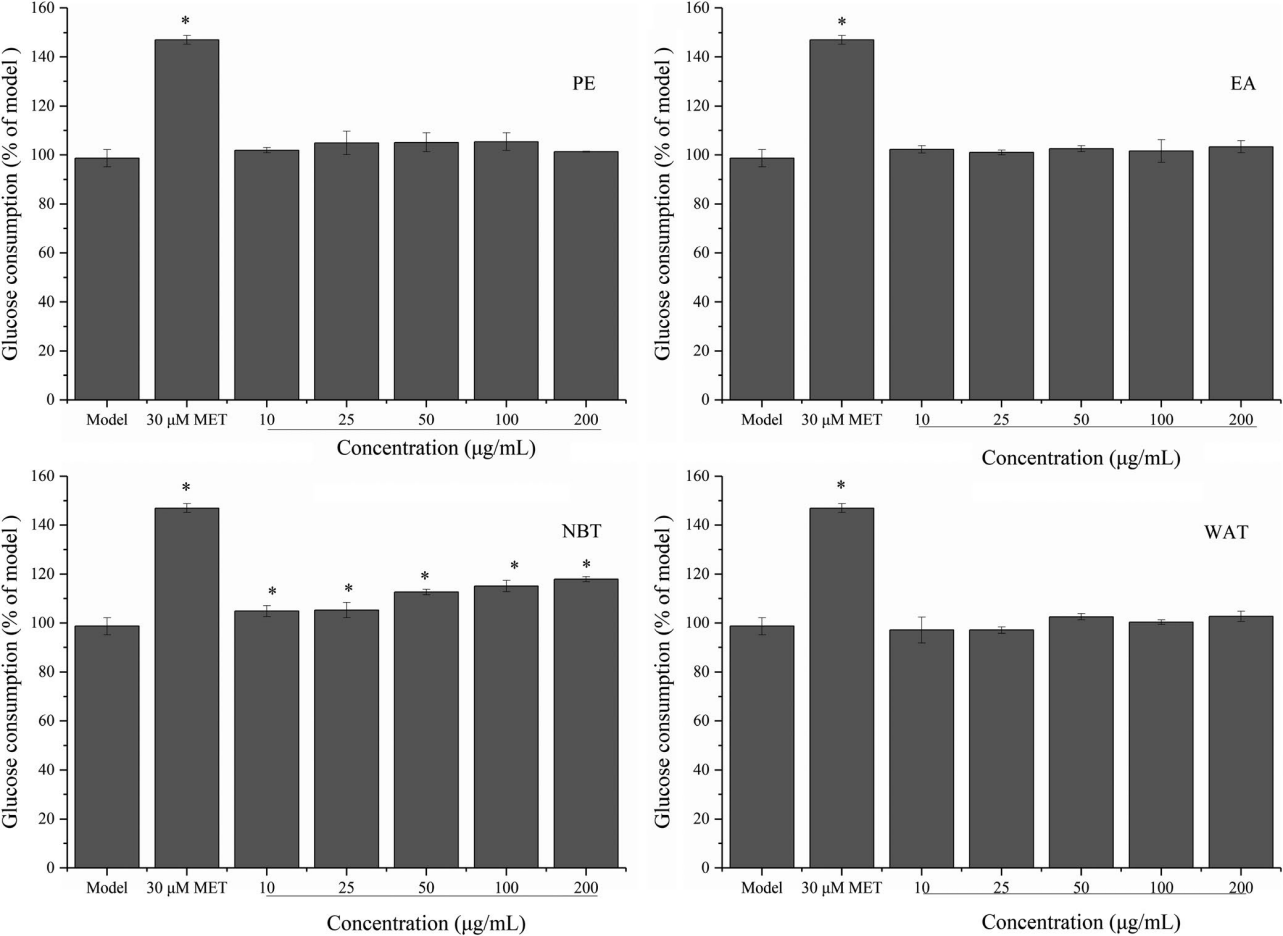
Subfractions of maca ethanol extract regulate glucose consumption in HepG2 cells. Subfractions: PE, petroleum ether; EA, ethyl acetate; NBT, n‐butanol; and WAT, water. *p < .05 compared with the model group

A previous study (Gonzales, 2015) indicated that maca ethanol extract could reduce glycemia in mice, which suggests that the extract might exhibit insulin‐mimetic activity to enhance glucose consumption at the cellular level. To understand the potential roles of maca extracts in glucose metabolism, this study first examined their ability to regulate glucose consumption in HepG2 model cells. As shown in Figure 7, the NBT fraction at 10, 25, 50, 100, and 200 μg/ml dose‐dependently and significantly increased glucose consumption by 8%, 8%, 18%, 20%, and 22% in HepG2 cells, respectively, compared with the results obtained with the model group. The effect was similar to that produced by MET (positive control). PE, EA, and WAT at 10–200 μg/ml did not exert any effect. A previous study showed that glucosinolates and flavonoids in maca could improve the glucose absorption of insulin‐resistant cells (Zhang, 2017). The NBT fraction might contain active ingredients such as glucosinolates and flavonoids.

**FIGURE 8:**
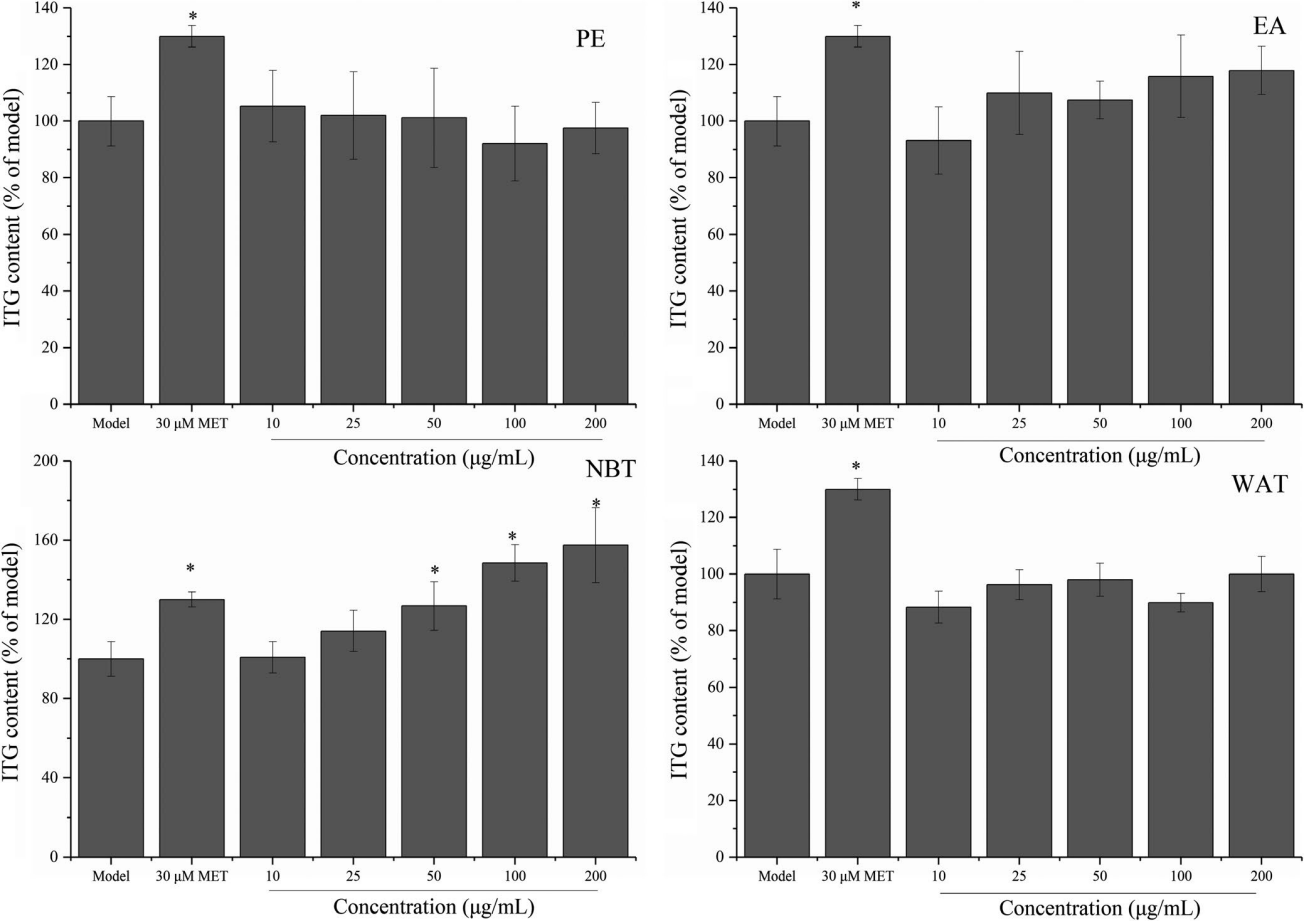
Subfractions of maca ethanol extract regulate the ITG levels in HepG2 cells. Subfractions: PE, petroleum ether; EA, ethyl acetate; NBT, n‐butanol; and WAT: water. *p < .05 compared with the model group

We also investigated the effect of subfractions of maca ethanol extract on 25G + 15F + 1FFA‐induced triglyceride accumulation. The ITG contents are presented in Figure 8. Treatment with the NBT fraction significantly increased the ITG contents compared with those found in the model group (*p* <.05), which suggested that the NBT fraction could promote the synthesis of TG in insulin‐resistant HepG2 cells. In particular, the NBT fraction increased the TG content in a dose‐dependent manner within 10–200 μg/ml. It is well known that insulin can significantly improve the metabolism of glucose and lipids in the human body by promoting glucose consumption and fat and protein synthesis in cells (Saltiel & Kahn, 2001). Therefore, the increase in TG after the NBT treatment might be related to the promotion of glucose absorption and lipid synthesis. Furthermore, the increase in lipids in cells can reduce the effect of glucotoxicity and thus improve insulin resistance in cells (Howard, 1999). We could conclude that the NBT fraction can improve insulin resistance by regulating glucose and lipid metabolism. Many chemically synthesized statin drugs could improve lipid metabolism and promote human health (Liu et al., 2018). However, the consumption of statin drugs for a long time can have some side effects, such as diarrhea, muscle aches or pain, nausea, indigestion, and weakness (Beltowski et al., 2009). Maca extracts, as a type of natural bioactive compound, might have fewer side effects and be developed as potential agents for regulating glucose and lipid metabolism.

**FIGURE 9:**
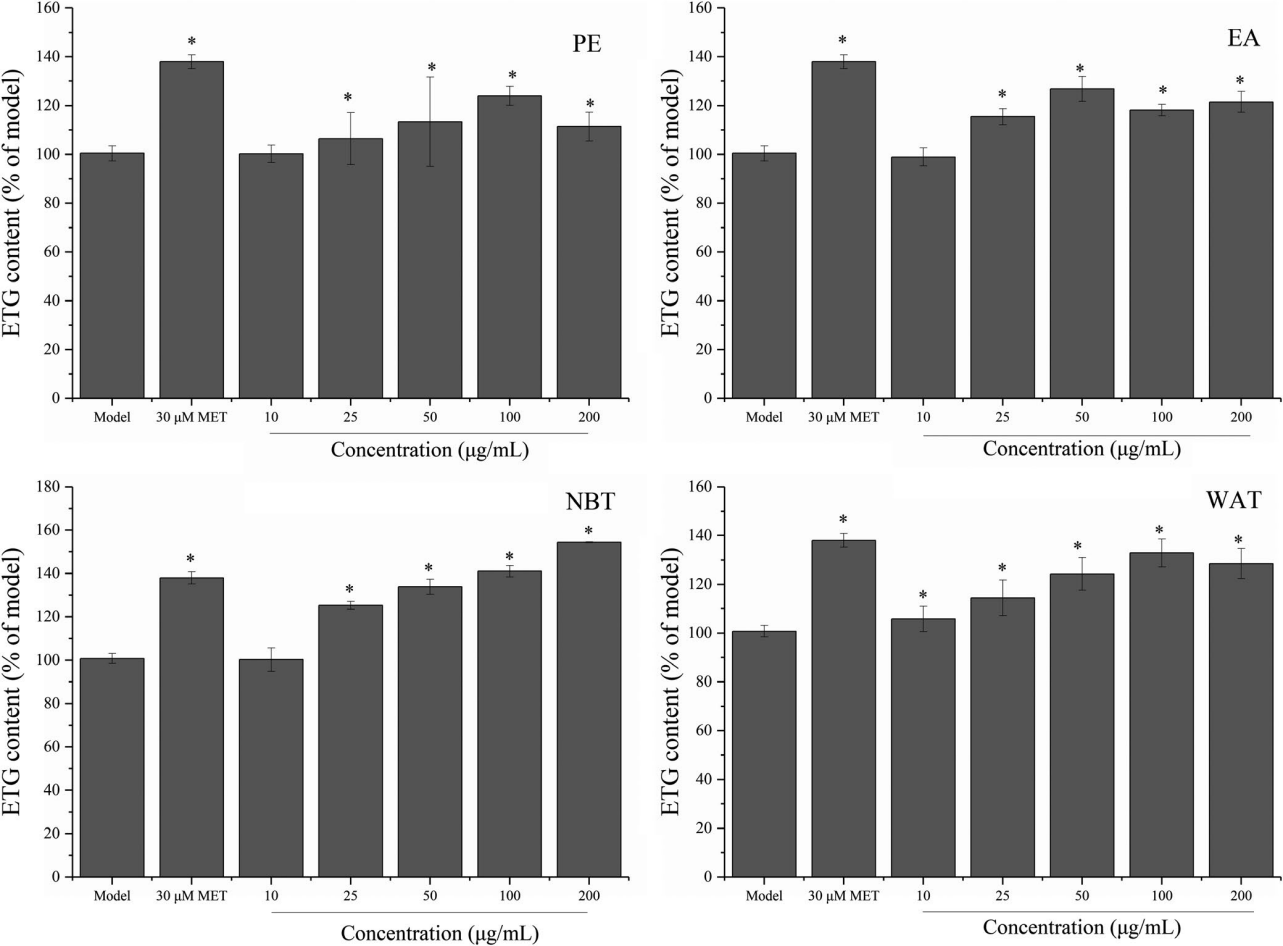
Subfractions of maca ethanol extract regulate the ETG levels in HepG2 cells. Subfractions: PE, petroleum ether; EA, ethyl acetate; NBT, n‐butanol; and WAT, water. *p < .05 compared with the untreated group

The ETG contents are presented in Figure 9. Treatment with maca extracts significantly increased the ETG contents in all four treated groups compared with that in the model group (*p* < .05), which suggested that maca ethanol extract could improve the secretion of TG in insulin‐resistant HepG2 cells. In particular, the NBT fraction at a concentration of 200 μg/ml increased the ETG content by approximately 50%, and this effect was better than that obtained with 30 μM metformin. Metformin can stimulate glucose transport and utilization in cells and promote the synthesis of triglycerides in cells (Lenhard et al., 1997); thus, the NBT fraction might exert a similar effect as MET on regulating glucose consumption.

### 3.6 LC‐MS/MS analysis

**FIGURE 10:**
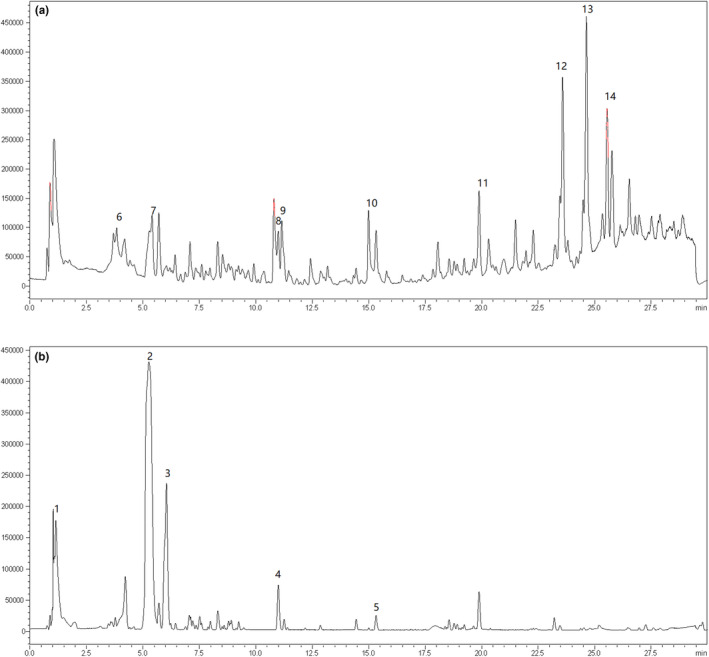
Total ion chromatogram of the NBT fraction determined by UPLC‐Q‐TOF MS: (a) positive mode and (b) negative mode

The phytochemical composition of the NBT fraction was further determined by LC‐MS/MS analysis in the positive mode. The total ion chromatogram (TIC) of the NBT fraction is shown in Figure 10. Supported by the fragment pattern (Table 2), the Spectral Database and previous reports (Long‐bo et al., 2017; Zhou et al., 2017), the corresponding compounds were initially determined. Eight representative peaks (Table 2) were tentatively identified as N‐(3,4‐dimethoxybenzyl)‐hexadecanamide (Wang, 2015), benzyl glucosinolate, (2R)‐2‐hydroxy‐2‐phenethylglucos, L‐phenylalanine, L‐tryptophan, N‐benzyl‐(9Z,12Z,15Z)‐octadecatrienamide), N‐benzyl‐(9Z,12Z)‐octadecadienamide; Xing Li & Chen, 2017), and N‐benzylhexadecanamide. The specific mass spectrum information is provided in the supplementary materials.

**TABLE 2:** Mass spectral data of the identified compounds in maca

Peak	RT (min)	[*M* + *H*]^+^ (*m*/*z*)	MS^2^ (*m*/*z*)	Molecular formula	Identification
1	1.053	404.10445	174.9552	C_23_H_67_NO_3_	*N*‐(3,4‐dimethoxybenzyl)‐hexadecanamide
2	5.274	408.07479	96.9595	C_14_H_19_O_9_NS_2_	Benzyl glucosinolate
3	6.051	438.05297	96.9596 138.9700 250.0121 438.0526	C_15_H_12_N_5_O_11_	(2R)−2‐Hydroxy−2‐phenethylglucos
4	11.003	263.08543	114.0555	C_5_H_11_N_8_O_5_	unknown
5	15.309	255.09544	91.0537, 121.0647, 255.1488	C_7_H_11_N_8_O_3_	unknown
6	3.826	166.0862	77.0383, 120.0806	C_9_H_11_NO_2_	L‐Phenylalanine
7	5.29	188.07072	91.0538, 115.0543,118.0653	C_12_H_13_NO	L‐Tryptophan
8	11.003	265.10045	70.0649 91.0540 116.0704	C_21_H_13_	unknown
9	11.174	307.18043	91.0539 121.0644 307.1804	C_20_H_23_N_2_O	unknown
10	15.322	257.11041	91.0539	C_7_H_13_N_8_O_3_	unknown
11	19.897	277.21594	77.0382 91.0539 106.0695 119.0856 121.0645 147.1167 277.2159	C_18_H_29_O_2_	unknown
12	23.592	368.29441	91.0539 108.0805 368.2945	C_25_H_37_NO	*N*‐benzyl‐(9Z, 12Z, 15Z)‐octadecatrienamide)
13	24.646	370.31003	91.0539 108.0805 190.1221 370.3096	C_25_H_39_NO	*N*‐benzyl‐(9Z, 12Z)‐octadecadienamide)
14	25.567	346.31007	91.0539 346.3099	C_23_H_39_NO	*N*‐benzylhexadecanamide

Note

1–5 refer to mass spectral data obtained in the negative mode; 6–14 show mass spectral data obtained in the positive mode.

### 3.7 Gene expression analyses of the PI3K/AKT and AMPK signalling pathways

**FIGURE 11:**
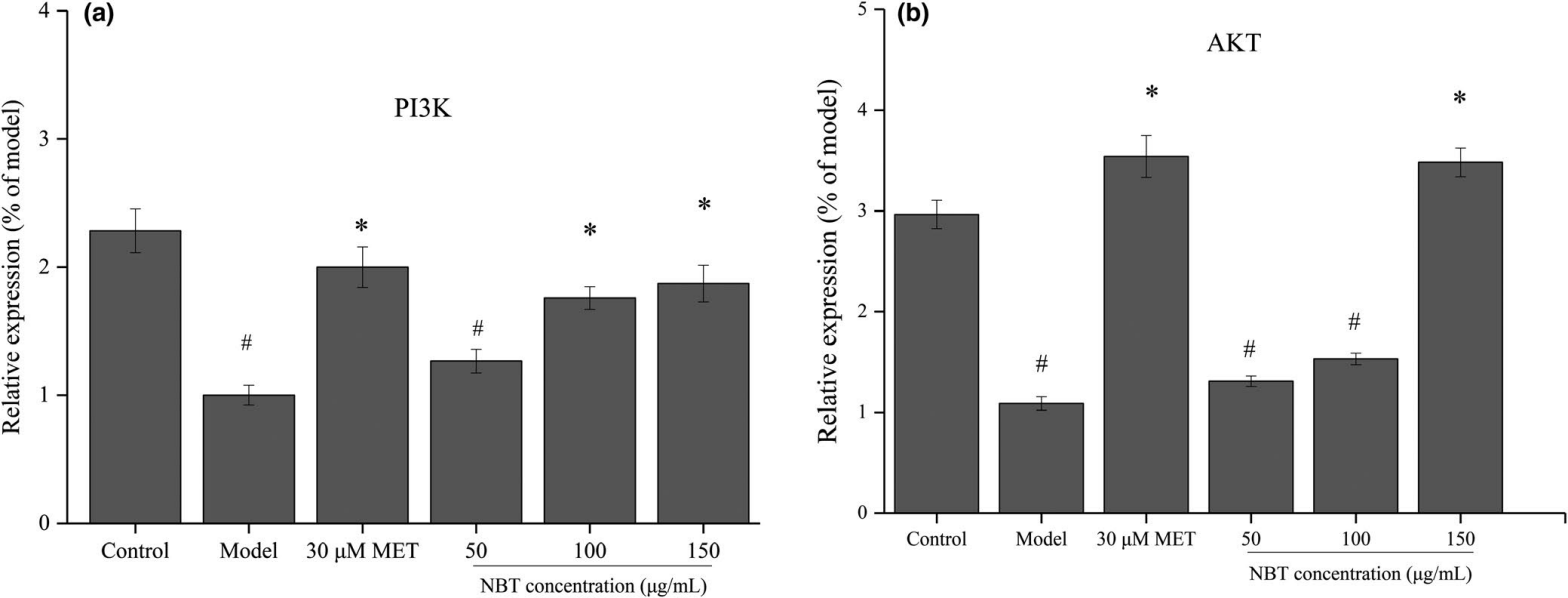
Effect of the NBT‐treated cells on the *PI3K* (a) and *AKT* (b) mRNA levels. ^#^ *p* < .05 compared with the control group, *p < .05 compared with the model group

It is well known that insulin plays an important role in glucose homeostasis by accelerating glucose transport and consuming energy via *PI3K*/*AKT* (Schultze et al., 2012). The activation of PI3K leads to the activation of *AKT*, which is a central mediator of many metabolic and growth actions of insulin. This kinase has been identified as one of the kinases responsible for the inactivation of glycogen synthase kinase through phosphorylation. The activation of Akt also promotes the translocation of GLUT4 vesicles to the plasma membrane (Abdul‐Ghani & Defronzo, 2010). As shown in Figure 11, the gene expression of *PI3K* and *AKT* was analyzed in HepG2 cells with glucose and lipid metabolic disorders. Compared with the control group, the model group showed significantly decreased *PI3K/AKT* gene expression levels, which suggests that 25G + 10F + 1FFA can induce serine/threonine phosphorylation of the insulin receptor INSR and thus reduce the ability to activate *P13K* and weaken the signal transduction of the downstream insulin receptor GLUT4, and these effects result in abnormal glucose metabolism. Compared with the results obtained with the model group, the mRNA expression of *PI3K* and *AKT* in the NBT‐treated group was positively related to the concentration of the NBT fraction (*p* < .05), which indicated that the NBT fraction could regulate cell glucose metabolism by activating the *PI3K* pathway to promote *AKT* expression. This conclusion was consistent with the results from the analysis of glucose consumption described in Section 3.5. We can thus conclude that the NBT fraction activated the *PI3K*/*AKT* signalling pathway by increasing insulin sensitivity.

## 4 DISCUSSION

Natural products reportedly have multiple biological activities. In China, many types of natural plants are used as functional food and for clinical treatment (Bagherniya et al., 2018; Lee et al., 2020), but the lack of a clear definition of the active constituents and mechanisms of action of natural plants have limited their application. Maca (*Lepidium meyenii* Walpers) has been used as a dietary supplement and ethnomedicine for centuries. Recently, maca has become a high‐profile functional food worldwide due to its multiple biological activities, such as enhancing fertility and sexual performance (Perrone et al., 2007) and showing antifatigue (Yue et al., 2019) and antioxidant activity (Vecera et al., 2007)^.^ Previous in vivo studies have shown that maca can significantly improve glucose tolerance and decrease the level of TAG (triacylglycerols) in plasma (Vecera et al., 2007). The aqueous extract of black maca could prevent and improve high‐fat, high‐fructose diet (HFD)‐induced metabolic disorders in golden hamsters (Wenting et al., 2018).

Maca contains alkaloids, glucosinolates, sterols and other active components. This study provides the first assessment of the in vitro activity of maca ethanol extract, and the specific active components were also identified. We first found that the NBT phase of maca ethanol extract could regulate glucose and lipid metabolism, and this effect might be due to the identified active components, such as benzyl glucosinolate, in the NBT fraction. A previous report also showed that glucosinolates might play an important role in glucose and lipid metabolism (Miao et al., 2012), which is consistent with our results. In addition, we also proved that the other subfractions of MEE, such as the PE, EA, and WAT fractions, have no positive effect on improving glucose and lipid metabolism.

Diabetes is a common and complex disease with a long course, multiple complications and hard‐to‐heal characteristics (Duarte et al., 2019). Previous studies have indicated that insulin participates in the metabolism of glucose and lipids, and insulin resistance could reduce glucose consumption in the cells and induce abnormal lipid levels (Peppa et al., 2015). Insulin‐mediated glucose uptake has been found to be reduced by more than 50% in patients with type 2 diabetes mellitus, which is related to specific alterations in the insulin signal transduction pathway, such as the PI3K/AKT and AMPK signalling pathways. Insulin resistance will result in reduced PI3K activity or altered protein expression of the regulatory subunit of PI3K. For instance, rosiglitazone increases insulin‐stimulated glucose transport via the PI3K signalling pathway by regulating the expression and activation of PI3K and AKT (Park et al., 2008). In addition, many active ingredients, including β‐sitosterol, mango tree leaf extracts, p‐coumaric acid and rosemary (*Rosmarinus officinalis* L.) extract, could improve glucose and lipids via the AMPK signalling pathway (Hwang et al., 2008; Yoon et al., 2013; Zhang et al., 2013).^1^ Wenting Wan found that aqueous extract of black maca could prevent metabolic disorders by regulating the glycolysis/gluconeogenesis‐TCA cycle and PPARα signalling activation (Li et al., 2018). In our study, we first investigated maca alcohol extract and identified the n‐butanol‐graded active components by tracking their activity. We then found that the NBT fraction could improve glucose and lipid metabolism in insulin‐resistant cells by regulating the APK and PI3K signalling pathways.

Many studies have researched the regulation of glucose and lipid metabolism by natural products, but few studies studied maca. Maca glucosinolate can exert hypoglycemic effects (Guzman‐Perez, 2014), but the mechanism is not very clear. Some studies have preliminarily proven that maca glucosinolates could regulate the insulin resistance status and then improve glucose metabolism (Zhang, 2017). Thus, the compounds in the NBT fraction identified in our study, namely, benzyl glucosinolate and (2R)‐2‐hydroxy‐2‐phenethylglucos, might be the main active ingredients that could increase insulin sensitivity and exhibit the above‐mentioned biological activity.

## 5 CONCLUSIONS

In conclusion, we performed the first investigation of the effects of maca extracts on glucose and lipid homeostasis in insulin‐resistant cells and the possible underlying mechanism involving the *PI3K*/*AKT* signalling pathway. The results showed that maca ethanol extracts exhibited significant antioxidant capacity. In addition, subfractions of maca ethanol extract could improve glucose and lipid metabolic disorders in HepG2 cells. In particular, the NBT phase of maca ethanol extract showed the most significant ability to improve glucose and lipid homeostasis. In addition, the active components in the NBT fraction were also identified. As an initial step to further investigate the mechanism, we partly revealed that the NBT phase of maca ethanol extract contained the active component benzyl glucosinolate and could improve glucose and lipid metabolism by upregulating *PI3K* and *AKT*.

## CONFLICT OF INTEREST

The authors declare no conflicts of interest.

## ETHICAL APPROVAL

This study does not involve any human or animal testing.

## Supporting information

Supplementary Material

## ENDNOTE

^1^ Rosemary (*Rosmarinus officinalis* L.) Extract Regulates Glucose and Lipid Metabolism by Activating AMPK and PPAR Pathways in HepG2 Cells.
