# Supplementary material for: Maca extracts regulate glucose and lipid metabolism in insulin‐resistant HepG2 cells via the PI3K/AKT signalling pathway
Source: Food Sci Nutr. 2021 Mar 29;9(6):2894–907. doi: 10.1002/fsn3.2246 (PMC8194906; doi:10.1002/fsn3.2246)
Supplement: Supplementary file 1 — Supplementary Material [file FSN3-9-2894-s001.doc]

ESI+

MS1 168.0478

MS1 188.0707

MS1 225.1385

MS1 277.1698

MS1 265.1005

MS1 307.1804

MS1 257.1104

MS1 277.2159

MS1 368.2944

MS1 370.3100

MS1 346.3100

MS1 372.3256

MS1 338.3412

ESI-

MS1 438.0529

MS1 263.0854
